# Effects of Nodulation on Metabolite Concentrations in Xylem Sap and in the Organs of Soybean Plants Supplied with Different N Forms

**DOI:** 10.3390/metabo13030319

**Published:** 2023-02-21

**Authors:** Takuji Ohyama, Miyuki Isaka, Akihiro Saito, Kyoko Higuchi

**Affiliations:** Department of Agricultural Chemistry, Faculty of Applied Biosciences, Tokyo University of Agriculture, Tokyo 156-8502, Japan

**Keywords:** nodule, allantoate, allantoin, asparagine, capillary electrophoresis, glutamine, nitrate, soybean, urea, xylem sap

## Abstract

The effects of nodulation on N metabolism in soybean plants supplied with various forms of N are not fully understood. Ureides are the principal forms of N transported from nodules, but nitrate and asparagine are the primary N compounds transported from roots supplied with NO_3_^−^. In this research, the effects of 1-day treatments of NO_3_^−^, NH_4_^+^, urea, or NO_3_^−^ + NH_4_^+^ on N metabolite concentrations in xylem sap and each organ were compared between nodulated and non-nodulated soybeans. Capillary electrophoresis and colorimetry were used for the analysis. In the xylem sap of the nodulated plants with an N-free solution, ureides were the major N metabolites, followed by asparagine and glutamine. Ureides concentrations were much lower in the xylem sap of the non-nodulated soybeans. In the NO_3_^−^ treatment, the concentrations of ureides in the xylem sap of the nodulated plants decreased compared to the control plants. In the NH_4_^+^, urea, and NO_3_^−^ + NH_4_^+^ treatments, the concentrations of asparagine and glutamine increased significantly compared with the control and NO_3_^−^ treatments. Similar changes with the N treatments were observed between the nodulated and non-nodulated soybeans, suggesting that nodulation does not have significant effects on the metabolism of absorbed N in roots.

## 1. Introduction

Soybean plants use nitrogen (N) fixed by root nodules, as well as the N absorbed from the roots. When soybean seeds were inoculated with rhizobia and grown in an N-free culture solution, small nodules, which were visible approximately 8 days after planting (DAP), started to fix N_2_ at approximately 17–19 DAP [1,2]. It has been well established that the initial assimilation product is NH_4_^+^, either due to the nitrogen fixation by nitrogenase or the NO_3_^−^ reduction by nitrate reductase (NR) and nitrite reductase (NiR) in roots and leaves [3]. However, the principal forms of N transport from nodules and roots are different [4,5]. Tracer experiments with ^15^N_2_ revealed that the ammonia produced by nitrogen fixation in bacteroid is rapidly released to the cytosol of infected cells and is initially assimilated into the amide group of glutamine (Gln) by the enzyme glutamine synthetase (GS). Then, Gln and 2-oxoglutarate produce two molecules of glutamate (Glu) by the enzyme glutamate synthase (GOGAT) [6,7]. Some parts of Gln are used for de novo purine synthesis in infected cells, and urate is transported from infected cells to the adjacent uninfected cells in the central symbiotic region of the nodule [8]. Urate is then catabolized into allantoin and allantoate in the uninfected cells and transported to the shoot through xylem vessels in the roots and stems. Ureide-transporting leguminous plants are limited to Phaseoleae, called tropical legumes, including *Glycine*, *Vigna*, and *Phaseolus* sp. However, the other legumes transport amides, especially asparagine (Asn) [8]. Ureides are the principal N transport compounds from soybean nodules, but Asn is also transported from the nodules. Huber and Streeter [9] reported that glutamine-dependent asparagine synthetase (AS) catalyzes the amidation of aspartate (Asp) to Asn in the cytosol fraction of the infected zone of soybean nodules. Minamisawa et al. [10] estimated that asparagine-N was approximately one-fifth of the ureide-N in the transport forms of fixed N from soybean nodules.

**Figure 1:**
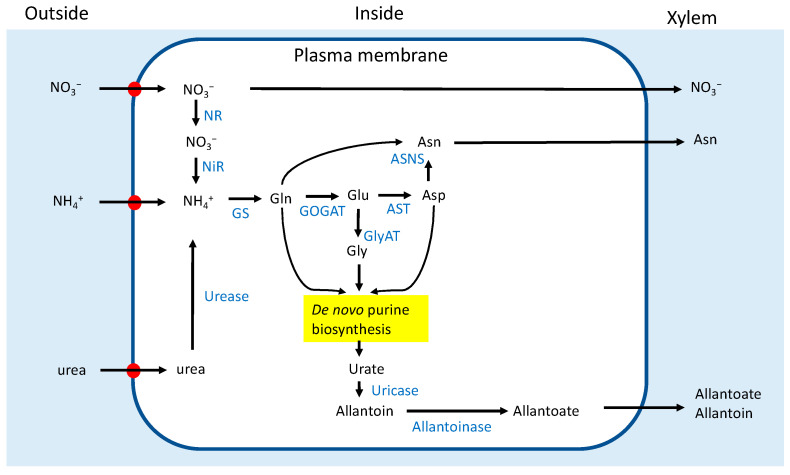
Schematic outline of the absorption and assimilation of NO_3_^−^, NH_4_^+^, and urea in a root cell. Transporters. ASNS: asparagine synthetase; AST: aspartate aminotransferase; GlyAT: glycine aminotransferase; GOGAT: glutamate synthase; GS: glutamine synthetase (referred to [3,15]).

NO_3_^−^ in the soil solution is absorbed by nitrate transporters located in the plasma membrane of the root cells (Figure 1). Some NO_3_^−^ is reduced in the roots to NO_2_^−^ by the enzyme NR and to ammonium by NiR. The other NO_3_^−^ is transported to the leaves through xylem vessels and then reduced to ammonium in the leaves. The ammonium produced by nitrate and nitrite reduction further assimilates into Gln and Glu by the GS/GOGAT cycle, similar to the assimilation of fixed N_2_ in the nodules; however, the major N transport form from the absorbed NO_3_^−^ in the roots is Asn synthesized by asparagine synthetase (AS). When a culture solution containing ^15^NO_3_^−^ was supplied to non-nodulated soybean plants for 2 h, the solution changed to unlabeled NO_3_^−^. Thereafter, the percentage of N derived from ^15^N increased rapidly during the initial 2h of the labeling period up to 60%; then, it decreased to 15% after 3h of the unlabeled chase period [11]. Ureides (allantoate and allantoin) are also produced in non-nodulated soybean roots, but the concentration in the xylem sap is approximately 10–30% of the sum of ureides, amides, and NO_3_^−^, much lower than those from nodules at approximately 80–90% [12]. The mechanisms by which ureide synthesis is dominant in nodules but not in roots have not been fully elucidated. A high concentration of ammonium may be related to the promotion of ureide synthesis [13]. It was suggested that N fixed in the nodules and N absorbed from the roots were differentially transported to the shoots [14,15]. The absorbed N, especially NO_3_^−^ is initially transported to the leave and assimilated there, and the N is redistributed to the pods and seeds. On the other hand, the N fixed is transported to both leaves and pods. So, the excess supply of N fertilizer sometimes causes over-luxuriant vegetative growth, but fewer pods. The difference may be due to the transport forms of N metabolites, so it is important to investigate how the nodulation and N supply affects the concentrations of principal transport forms of N.

In addition to NO_3_^−^, soybean roots can efficiently absorb and assimilate NH_4_^+^, urea, and Gln in solution [2]. Figure 1 shows an outline of the metabolic pathways for the assimilation of NO_3_^−^, NH_4_^+^, and urea. Ono et al. [16] investigated the effect of the application of 5 mM-N of NO_3_^−^, NH_4_^+^, or urea to nodulated soybean plants for 3 days on the concentrations of N metabolites in the xylem sap and each organ. The ureide concentration in the xylem sap was the highest in the control plants supplied with an N-free nutrient solution. However, nitrate and asparagine were the principal compounds in the xylem sap with nitrate treatment. Under NO_3_^−^ treatment, the concentration of ureides in the xylem sap significantly decreased to one-seventh of the control, while it decreased to one-half under NH_4_^+^ and urea treatments.

In this experiment, the concentrations of principal N metabolites in the xylem sap and each organ of nodulated and non-nodulated soybean were compared after 1 d treatments of 5 mM-N of NO_3_^−^, NH_4_^+^, urea, or a mixture of NO_3_^−^ + NH_4_^+^. The nodulated soybean plants were cultivated with an N-free culture solution in which soybean plants depended only on N_2_ fixation. To support the N requirement of the non-nodulated soybean, 1 mM of NO_3_^−^ was supplied to the culture solution until 27 DAP. The plants were cultivated in N-free solution for 3 days from 27 to 30 DAP to reduce the NO_3_^−^ and related N metabolites stored in the plants. After the plants were treated with various forms of N for 24 h, xylem sap was collected from the cut basal stem for 1 h. The roots were separated into upper and lower parts. Most of the large active nodules were distributed on the upper part of the roots, and nodules on the lower roots were scarce and small if present. The effects of nodulation on the N absorption, assimilation, and transport of N metabolites in soybean roots supplied with various forms of N have not been evaluated. The concentrations of principal N metabolites in the xylem sap and organs between nodulated and non-nodulated plants were compared in this study.

## 2. Materials and Methods

### 2.1. Plant Cultivation and Nitrogen Treatments

Soybean plants (*Glycine max* (L.) Merr., cultivar “Williams”) were cultivated in a biophotochamber (LH-350S, Nippon Medical & Chemical Instruments Co., Ltd., Osaka, Japan) at 28 °C day/18 °C night temperatures, 55% relative humidity, and under a photoperiod of 16 h light (228 µmol photons m^−2^ s^−1^)/8 h darkness. The nodulated (Nod) plants were cultivated after the seeds were inoculated with a suspension of *Bradyrhizobium diazoefficiens* (USDA 110). The non-nodulated (Non-nod) plants were cultivated in a separate chamber from the nodulated plants to prevent rhizobial infection. At 10 days after planting (DAP), the nodulated plants (Nod) were transplanted into 800 mL of nitrogen-free nutrient solution [16] in a 900 mL glass bottle with continuous aeration. The glass bottle was covered with aluminum foil for shading the culture solution and roots. The noninoculated plants were cultivated in the same manner with a culture solution containing 1 mM NaNO_3_ until 27 DAP to support the N demand without nitrogen fixation. The culture solutions were renewed every 3 days. The N-free solution was supplied from 27 to 30 DAP right before the N treatments to lower the levels of NO_3_^−^ and related N metabolites in the non-nodulated plants. At 30 DAP, both the Nod and Non-nod plants were treated with different N sources in a culture solution in which the N concentrations were adjusted at 5 mM-N. The N treatments were as follows: Cont, N-free solution; NO_3_^−^, 5 mM KNO_3_; NH_4_^+^, 2.5 mM (NH_4_)_2_SO_4_; urea, 2.5 mM urea; and NO_3_^−^ + NH_4_^+^, 2.5 mM KNO_3_ + 1.25 mM (NH_4_)_2_SO_4_. After 24 h of the N treatments at 31 DAP, the basal part of the stem was cut using a razor blade, and the xylem sap was collected for 1 h in a tube connected to the basal part of the main stem [17]. The xylem sap was collected from the cut end of the basal stem, and it consisted of xylem transport compounds from the roots to the shoots.The plant roots were washed and separated into the nodules and the upper and lower parts of the roots. The shoots and roots of the plants were dried using a freeze dryer (VD-400F, TAITEC, Saitama, Japan) and separated into leaves, stems plus petioles, upper and lower roots, and nodules. Then, the dry samples were ground into a fine powder.

### 2.2. Analysis of the Principal N Metabolites

Approximately 25 mg of ground sample powder was extracted with 1 mL of 80% ethanol containing 0.2 mM MES (2-(N-morpholino)ethanesulfonic acid) as an internal standard for capillary electrophoresis [18]. Then, the ethanol extract was evaporated in a vacuo and redissolved in water. Then, the concentrations of nitrate, Glu, Asp, Gln, Asn, allantoin, and allantoate in the extract and in the xylem sap were analyzed by capillary electrophoresis (7100, Agilent Technologies, Inc., Santa Clara, CA, USA) using a fused silica tube (inner diameter (id): 50 μm; length: 104 cm) and a commercial buffer solution (α-AFQ109, Ohtsuka Electronics Co., Ltd., Osaka, Japan), with an applied voltage of −25 kV. Peaks were detected with a signal wavelength of 400 nm and a reference wavelength of 265 nm.

The ammonium concentration was determined by the indophenol method using a microplate reader [19]. The reagents were prepared as follows. 2,2′,2″,2′′′-(Ethane-1,2-diyldinitrilo)tetraacetic acid (EDTA) solution: dissolve 2.5 g of EDTA·2Na in 80 mL of water, adjust the pH to 10, and then fill to 100 mL. An amount of 1 M phosphate buffer: Dissolve 13.61 g of KH_2_PO_4_ and 0.275 g of benzoic acid in 100 mL of water. Nitroprusside solution: Mix 10 mg of sodium nitrosylpentacyanoferrate(III) dihydrate, 1.025 mL of liquid phenol (mix 47 mL of water in 500 g of melted phenol by warming in a water bath), and 80 mL of water. Then, fill to 100 mL, followed by filling to 1000 mL with water. Sodium hypochlorite solution: Dissolve 1 mL of sodium hypochlorite solution, 1 g of NaOH, 0.706 g of Na_2_HPO_4_, and 3.18 g of Na_3_PO_4_·12H_2_O in 80 mL of water. Fill to 100 mL with water. An amount of 1 M NaOH solution: Dissolve 4 g of NaOH in 100 mL water. Procedures: Put 10 μL of xylem sap or extracted solution in a 1.5 mL plastic tube, add 5 μL of EDTA solution, and stir. Second, add 5 μL of 1 M phosphate buffer and stir. Next, add 65 μL of nitroprusside solution and stir. Add 65 μL of sodium hypochlorite solution and stir. Finally, add 100 μL of water and stir. Let sit for 3 h in the dark until measurement. Measurement by microplate reader: Transfer 200 μL of the reaction solution into the well of a microplate (Watson, 96 holes with a flat bottom). Set the microplate in the microplate reader (S H-1000, Corona Electric, Co., Ltd., Ibaraki, Japan) and read the absorbance at 625 nm.

The urea concentration was calculated by subtracting the ammonium concentration after the urease reaction (ammonium + urea) from the ammonium concentration without the urease reaction [19]. Reagents: Glycerin stock of the urease solution: Dissolve 20 mg of urease (urease from Jack bean, Wako chemical, Japan) and 20 mg of EDTA·2Na solution in 50 mL of water. Add 50 mL of glycerin and stir; store in a freezer. Urease–phosphate buffer: mix 5 mL of urease glycerin stock and 5 mL of 0.2 M phosphate buffer (pH 6.0). Procedures for the urea + ammonium N concentration: Place 10 μL of xylem sap or the extracted water solution in a 1.5 mL plastic tube, add 100 μL of the urease–phosphate buffer, stir, and incubate at 37 °C for 15 min. Add 200 μL of the phenol nitroprusside solution (2.5 mg of sodium nitroprusside dissolved in 25 mL of 1% phenol water), stir, and incubate at 37 °C for 20 min. Transfer the 200 μL reaction mixture from the plastic tube into the well of a microplate, and read the absorbance at 625 nm using a microplate reader (SH-1000, Corona Electric Co., Ltd., Ibaraki, Japan). Use another 10 μL of the xylem sap or water extract for the ammonium analysis. Mix the sample solution in a plastic tube with 100 μL of phosphate buffer without urease. Next, add 200 μL of the phenol nitroprusside solution, and incubate the mixture at 37 °C for 20 min. Then, add 200 μL of alkaline hypochlorite reagent. Determine the absorbance at 625 nm the same as for the urea analysis above. The urea concentration was calculated by subtracting the ammonium concentration after the urease reaction (ammonium + urea) from that without the urease reaction (ammonium only).

### 2.3. Statistics

The experiments were conducted with 3 biological replications. The plants with the N treatments were randomly arranged in a growth chamber. To evaluate the statistical significance between the Nod and Non-nod plants, the Student’s *t*-test for the independent samples was used. Asterisks (*, **) designate significant changes according to Student’s *t*-test results for the same compound in the same treatment between nodulated and non-nodulated plants at *p* < 0.05 and *p* < 0.01, respectively. Tukey’s test was used for evaluating the statistical significance among the N treatments. The different letters on the top of the columns indicate significant differences in the N concentration among the treatments based on Tukey’s test (*p* < 0.05). The statistical significance was determined using the statistical analysis program of Osaka University.

## 3. Results

### 3.1. Concentrations of N Metabolites in the Xylem Sap

**Figure 2:**
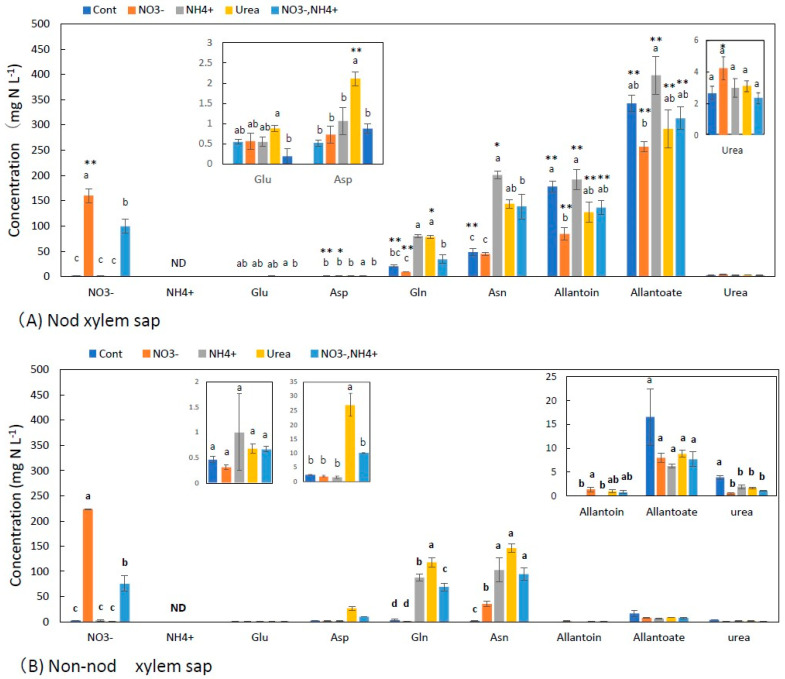
Concentrations of the principal N metabolites in the xylem sap of nodulated (**A**) and non-nodulated (**B**) soybean plants treated with various N compounds. Treatments: Cont, control with N-free solution; NO_3_^−^, 5 mM KNO_3_; NH_4_^+^, 2.5 mM (NH_4_)_2_SO_4_; urea, 2.5 mM urea; NO_3_^−^ + NH_4_^+^, 2.5 mM KNO_3_ + 1.25 mM (NH_4_)_2_SO_4_. *n* = 3. Average ± standard error. The different letters on the top of the columns indicate significant differences in the N concentration among the treatments based on Tukey’s test (*p* < 0.05). Asterisks (*, **) designate significant changes according to Student’s *t*-test results for the same compound in the same treatment between nodulated and non-nodulated plants at *p* < 0.05 and *p* < 0.01, respectively. ND; not detected.

Figure 2A,B show the concentrations of the principal N metabolites in the xylem sap of the nodulated and non-nodulated soybean plants. In the control treatment, the Nod plants that depended only on nitrogen fixation, the concentrations of allantoate (343 mg N L^−1^) and allantoin (178 mg N L^−1^) were the highest, followed by Asn (48 mg N L^−1^), Gln (20 mg N L^−1^), and urea (2.6 mg N L^−1^). The concentrations of Glu (0.55 mg N L^−1^) and Asp (0.52 mg N L^−1^) were low, and ammonium was not detected. The nitrate treatment increased the NO_3_^−^ concentrations at 160 mg N L^−1^ and decreased the allantoate and allantoin concentrations. The concentrations of Gln, Asn, Glu, and Asp did not significantly change with the nitrate treatment compared with the control. The NH_4_^+^ and urea treatments resulted in similar metabolite profiles in which the concentrations of Asn and Gln increased several times compared with the control xylem sap, and the concentrations of allantoate and allantoin were not significantly different from the control xylem sap. The 2.5 mM NO_3_^−^ + 2.5 mM NH_4_^+^ treatment showed an increase in NO_3_^−^, Asn, and Gln, but the values were lower than those in the 5 mM NO_3_^−^ treatment.

Figure 2B shows the concentrations of N metabolites in the xylem sap of the Non-nod soybean plants. The concentrations of metabolites in the Cont treatment were low, where the allantoate was 17 mg N L^−1^, urea was 3.9 mg N L^−1^, Gln 3.8 was mg N L^−1^, Asp was 2.4 mg N L^−1^, Asn was 2.0 mg N L^−1^, and Glu was 0.5 mg N L^−1^. Allantoin and ammonium were not detected. The low concentrations of the N metabolites in the Cont treatment might be due to the fact of N starvation for 4 days from 27 to 31 DAP, including the N-free cultivation period from 27 to 30 DAP and the Cont treatment from 30 to 31 DAP. The NO_3_^−^ concentration was 0.35 mg N L^−1^, suggesting that NO_3_^−^ stored in the roots might be depleted. The concentrations of allantoate and allantoin were significantly (*p* < 0.01) different among the Nod (A) and Non-nod (B) xylem sap. In the NO_3_^−^ treatment, the concentrations of nitrate (224 mg N L^−1^) and Asn (35 mg N L^−1^) increased, but the other compound did not. By the application of 5 mM-N of NH_4_^+^, urea, and NO_3_^−^ + NH_4_^+^, the concentrations of Asn and Gln were significantly increased compared with the Cont and NO_3_^−^ treatments. The application of the N forms did not affect the concentrations of allantoate, allantoin, and urea in the Non-nod xylem sap. Ammonium could not be detected in the xylem sap collected from all treatments, including the NH_4_^+^ treatment. The concentrations of allantoate and allantoin were significantly (*p* < 0.01) different in the Nod xylem sap compared with that of the Non-nod, indicating that a large portion of the ureides (allantoate and allantoin) in the xylem sap of the Nod derived from nodules, although small amounts of ureides in the xylem sap of the Non-nod plants might have come from the roots.

### 3.2. Concentrations of N Metabolites in the Nodules

**Figure 3:**
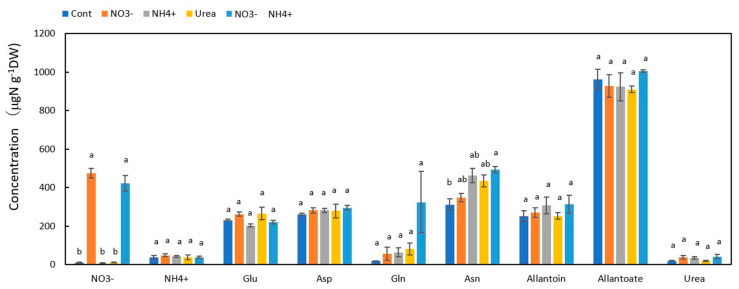
Comparison of the concentrations of the principal N metabolites in the nodules of nodulated soybean plants treated with various N compounds. Treatments: Cont, control with N-free solution; NO_3_^−^, 5 mM KNO_3_; NH_4_^+^, 2.5 mM (NH_4_)_2_SO_4_; urea, 2.5 mM urea; NO_3_^−^ + NH_4_^+^, 2.5 mM KNO_3_ + 1.25 mM (NH_4_)_2_SO_4_. *n* = 3. Average ± standard error. The different letters on the top of the columns indicate significant differences in the N concentration among the N treatments based on Tukey’s test (*p* < 0.05).

Among the N metabolites in the Cont nodules Figure 3, the concentration of allantoate was the highest at 963 μg N g^−1^DW, followed by Asn (311 μg N g^−1^DW), Asp (262 μg N g^−1^DW), allantoin (252 μg N g^−1^DW), and Glu (231 μg N g^−1^DW). In the NO_3_^−^ and NO_3_^−^ + NH_4_^+^ treatments, the NO_3_^−^ concentrations in the nodules were 476 and 422 μg N g^−1^DW, indicating that NO_3_^−^ accumulated in the nodules when NO_3_^−^ was supplied for 1 day. The concentrations of the other N metabolites in the nodules were not significantly affected by the N treatments.

### 3.3. Concentrations of N Metabolites in the Lower Roots

**Figure 4:**
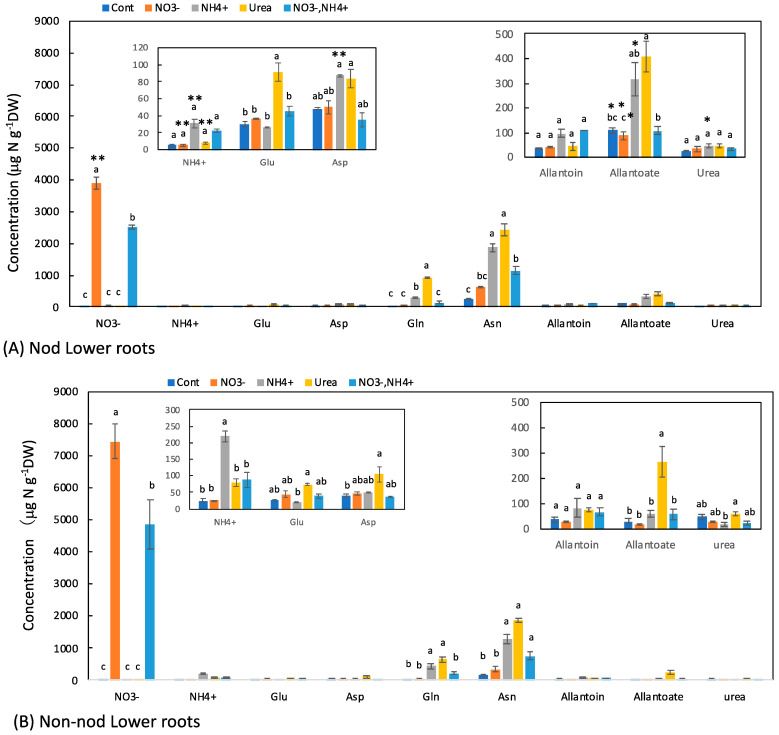
Comparison of the concentrations of the principal N metabolites in the lower roots of the Nod and Non-nod soybean plants treated with various N compounds. The symbols, abbreviations and the explanations of the statistics are the same as in Figure 2.

Most of the large nodules were attached to the upper part of the roots, and a few nodules were on the lower part of the roots. The concentrations of the principal N metabolite in the lower roots of the Nod and Non-nod plants were relatively similar (Figure 4A,B). In the Nod plants, the average concentration of Asn (238 mg N g^−1^DW) was the highest, followed by allantoate (109 μg N g^−1^DW), Asp (49 μg N g^−1^DW), allantoin (36 μg N g^−1^DW), Gln (30 μg N g^−1^DW), Glu (30 μg N g^−1^DW), and urea (26 μg N g^−1^DW). In the Non-nod plants, the average concentration of Asn (172 μg N g^−1^DW) was the highest, followed by allantoate (28 μg N g^−1^DW), Asp (39 μg N g^−1^DW), allantoin (37 μg N g^−1^DW), Gln (29 μg N g^−1^DW), Glu (25 μg N g^−1^DW), and urea (46 μg N g^−1^DW). The NO_3_^−^ concentrations in the Non-nod lower roots supplied with 5 mM NO_3_^−^ were higher (7450 μg N g^−1^DW) than that of the Nod lower roots (3900 μg N g^−1^DW). The application of NO_3_^−^ + NH_4_^+^ resulted in a lower NO_3_^−^ accumulation compared with 5 mM NO_3_^−^. The 5 mM NO_3_^−^ treatment tended to increase the Asn concentration but did not affect the other N metabolites, including allantoate and allantoin. On the other hand, the NH_4_^+^ and urea treatments markedly increased the concentrations of Asn, Gln, and allantoate in the lower roots.

### 3.4. Concentrations of N Metabolites in the Upper Roots

**Figure 5:**
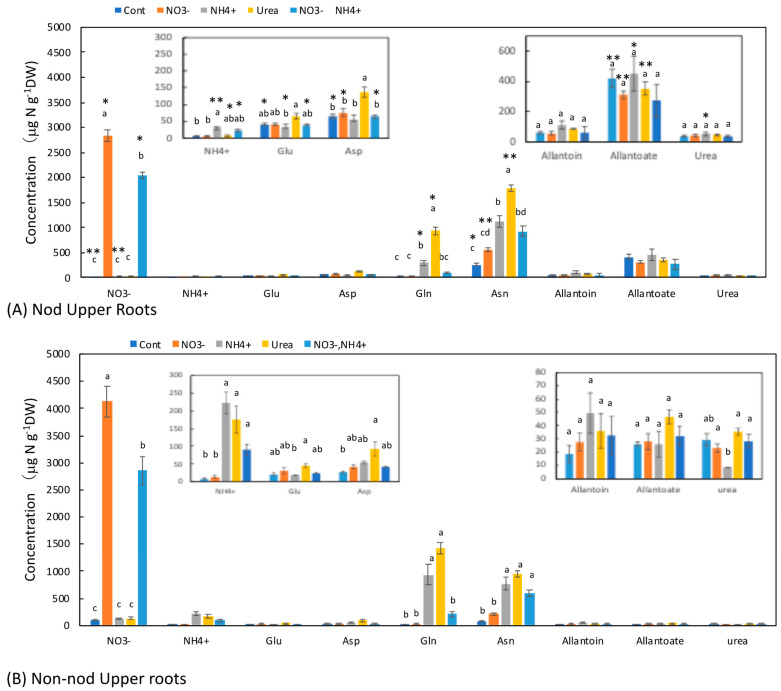
Comparison of the concentrations of the principal N metabolites in the upper roots of the Nod and Non-nod soybean plants treated with the various N compounds. The symbols, abbreviations and the explanations of the statistics are the same as in Figure 2.

The upper part of the Nod roots was a transport pathway for the fixed N from the nodules; thus, the concentrations of the N metabolites contained N metabolite from the nodules to the shoot, whereas the Non-nod upper roots did not have nodules. In the Nod plants (Figure 5A), the average concentration of allantoate (421 μg N g^−1^DW) was the highest, followed by Asn (260 μg N g^−1^DW), Asp (66 μg N g^−1^DW), allantoin (60 μg N g^−1^DW), Glu (42 μg N g^−1^DW), urea (38 μg N g^−1^DW), and Gln (28 μg N g^−1^DW). In the Non-nod plants (Figure 5B), the average concentration of Asn (85 mg N g^−1^DW) was the highest, followed by urea (29 μg N g^−1^DW), Asp (27 μg N g^−1^DW), allantoate (26 μg N g^−1^DW), Glu (21 μg N g^−1^DW), allantoin (18 μg N g^−1^DW), and Gln (17 μg N g^−1^DW). The concentration of allantoate in the Nod upper roots was significantly higher than that in the Non-nod upper roots, and the difference might have been due to the ureides deriving from nodules. The NO_3_^−^ concentrations in the Non-nod upper roots supplied with 5 mM NO_3_^−^ were significantly higher (4130 μg N g^−1^DW) than that of the Nod upper roots (2830 μg N g^−1^DW). The application of NO_3_^−^ + NH_4_^+^ resulted in lower NO_3_^−^ accumulation in the upper roots compared with 5 mM NO_3_^−^. The 5 mM NO_3_^−^ treatment tended to increase the Asn concentration, but this did not affect the other N metabolites, including allantoate and allantoin. On the other hand, the NH_4_^+^, urea, and NO_3_^−^ + NH_4_^+^ treatments markedly increased the concentrations of Asn and Gln in the upper roots. The profiles of the N metabolite concentrations were relatively similar between the upper and lower roots, except for the allantoate concentrations in the upper roots.

### 3.5. Concentrations of N Metabolites in the Stems

**Figure 6:**
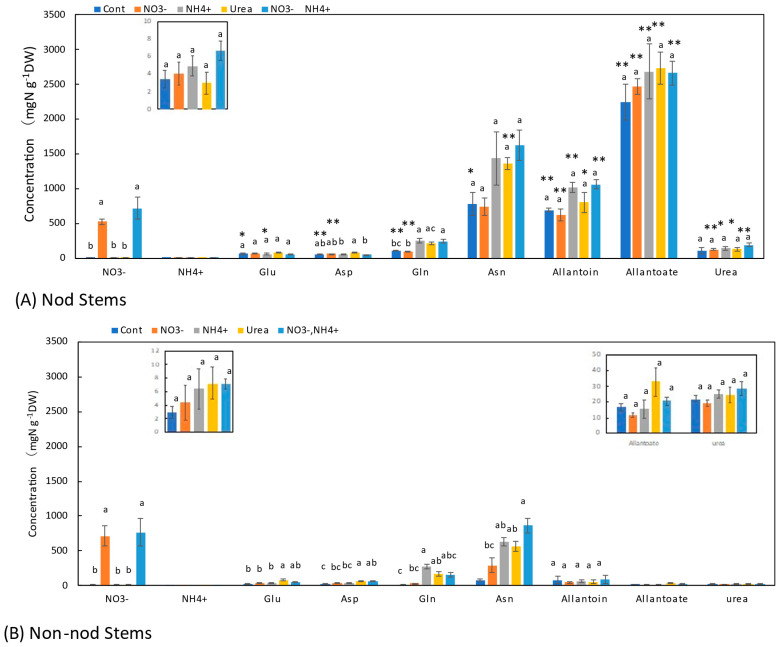
Comparison of the concentrations of the principal N metabolites in the stems of the Nod and Non-nod soybean plants treated with the various N compounds. The symbols, abbreviations and the explanations of the statistics are the same as in Figure 2.

In the stems of the Nod plants, the average concentrations of allantoate (2240 μg N g^−1^DW) were the highest, followed by Asn (785 μg N g^−1^DW), allantoin (691 μg N g^−1^DW), urea (112 μg N g^−1^DW), Gln (108 μg N g^−1^DW), Glu (66 μg N g^−1^DW), and Asp (61 μg N g^−1^DW). In the stems of the Non-nod plants, the average concentrations of allantoin (82 μg N g^−1^DW) and Asn (74 mg N g^−1^DW) were the highest, followed by Asp (23 μg N g^−1^DW), urea (22 mg N g^−1^DW), Glu (21 μg N g^−1^DW), allantoate (17 μg N g^−1^DW), and Gln (16 μg N g^−1^DW). The concentrations of allantoate and allantoin in the Nod stems were significantly (*p* < 0.01) higher than those in the Non-nod stems. The difference in the ureides concentration might be due to the nodules. The NO_3_^−^ concentrations between the Nod and Non-nod stems supplied with 5 mM NO_3_^−^ were not significantly different. The application of NO_3_^−^ + NH_4_^+^ resulted in lower NO_3_^−^ accumulation compared with 5 mM NO_3_^−^. The 5 mM NO_3_^−^ treatment tended to increase the concentration of Asn but not the other N metabolites, including allantoate and allantoin in the Nod and Non-nod stems. The NH_4_^+^, urea, and NO_3_^−^ + NH_4_^+^ treatments tended to increase the concentrations of Asn and Gln in the stems as in the upper and lower roots. The concentrations of allantoate, allantoin, and urea were significantly higher in the stems of the Nod plants than in the Non-nod plants (Figure 6).

### 3.6. Concentrations of N Metabolites in the Leaves

**Figure 7:**
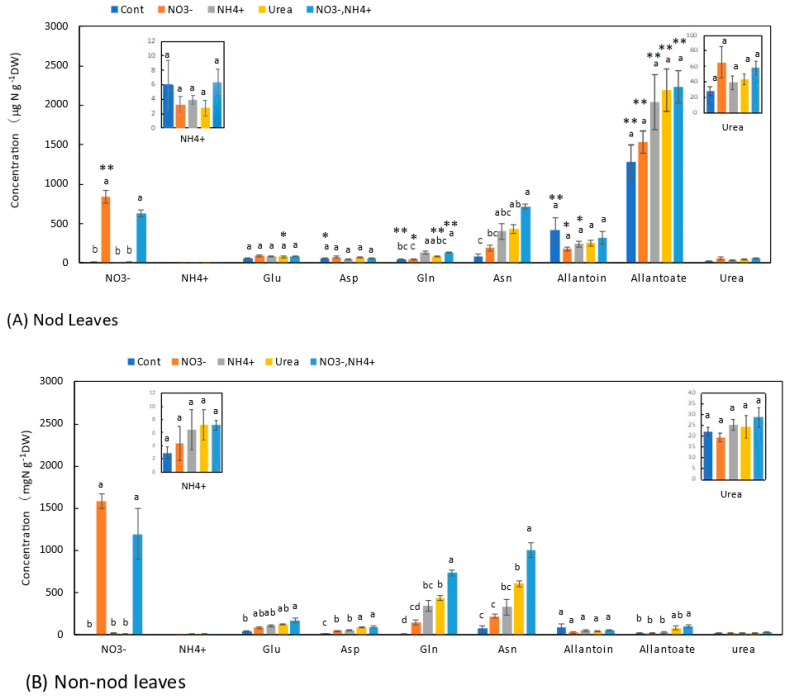
Comparison of the concentrations of the principal N metabolites in the leaves of the Nod and Non-nod soybean plants treated with various N compounds. The symbols, abbreviations and the explanations of the statistics are the same as in Figure 2.

Similar trends were observed in the leaves (Figure 7) and stems (Figure 6). In the leaves of the Nod plants with the Cont treatment, the average concentration of allantoate (1280 μg N g^−1^DW) was the highest, followed by allantoin (415 μg N g^−1^DW), Asn (87 μg N g^−1^DW), Glu (57 μg N g^−1^DW), Asp (57 μg N g^−1^DW), Gln (50 μg N g^−1^DW), and urea (28 μg N g^−1^DW). In the leaves of the Non-nod plants, the average concentrations of allantoin (85 μg N g^−1^DW) and Asn (82 mg N g^−1^DW) were the highest, followed by Glu (47 μg N g^−1^DW), urea (22 mg N g^−1^DW), allantoate (22 μg N g^−1^DW), Asp (17 μg N g^−1^DW), and Gln (11 μg N g^−1^DW). The concentrations of allantoate and allantoin in the Nod leaves were significantly higher than those in the Non-nod leaves. The difference in the ureides concentrations might have derived from the nodules. The NO_3_^−^ concentrations in the leaves of the Non-nod leaves supplied with 5 mM NO_3_^−^ were significantly (*p* < 0.01) higher (1580 μg N g^−1^DW) than in the Nod leaves (842 μg N g^−1^DW). The application of NO_3_^−^ + NH_4_^+^ resulted in a lower NO_3_^−^ accumulation compared with 5 mM NO_3_^−^. The 5 mM NO_3_^−^ treatment tended to increase the Asn concentration but did not affect the other N metabolites, including allantoate and allantoin in the Nod and Non-nod stems. The NH_4_^+^, urea, and NO_3_^−^ + NH_4_^+^ treatments increased the concentrations of Asn and Gln in the leaves. The concentrations of allantoate and allantoin were significantly higher in the leaves of the Nod plants than in the Non-nod plants (Figure 7).

### 3.7. Comparison of the Concentrations of N Metabolites among the Organs with N Treatments

**Figure 8:**
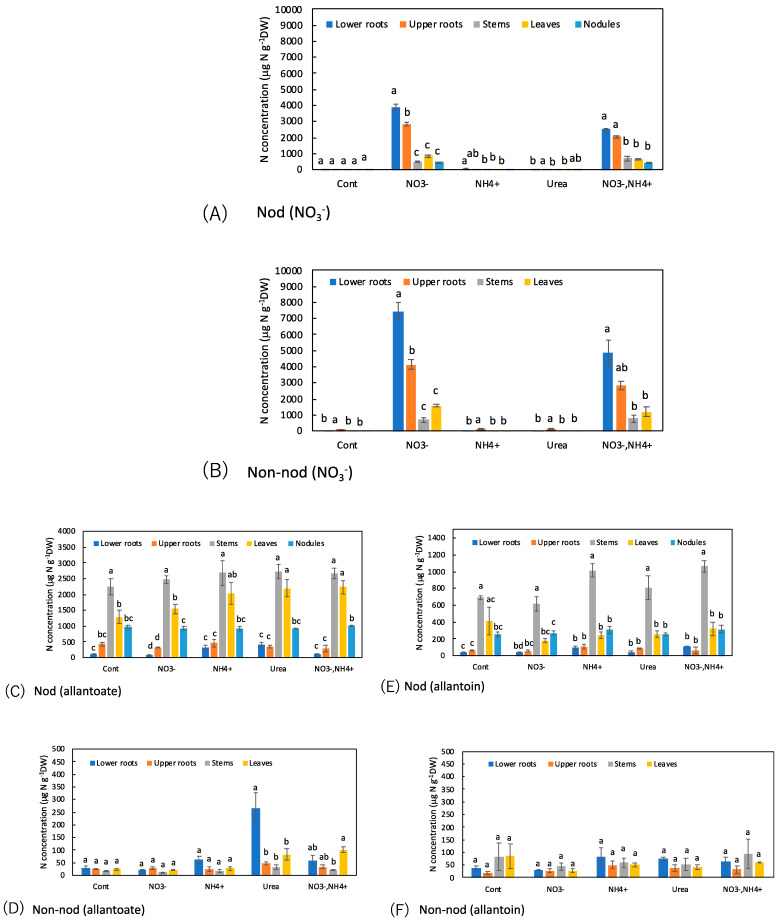

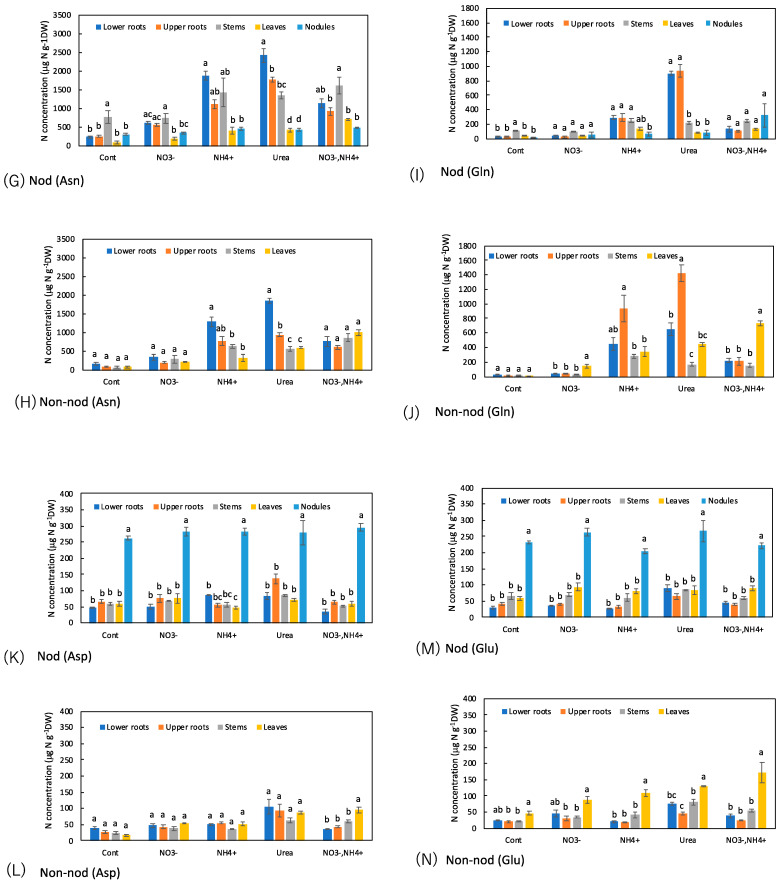

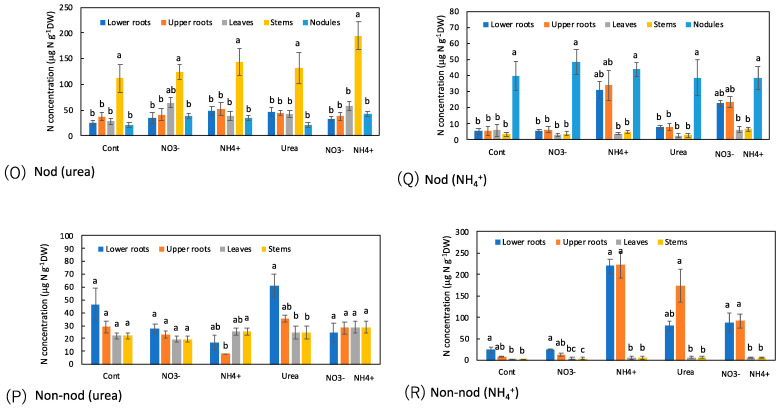
Comparison of the concentrations of N metabolites among the organs of the Nod and Non-nod soybean plants treated with the various N compounds: (**A**,**B**) NO_3_^−^; (**C**,**D**) allantoate; (**E**,**F**) allantoin; (**G**,**H**) Asn; (**I**,**J**) Gln; (**K**,**L**) Asp; (**M**,**N**) Glu; (**O**,**P**) urea; (**Q**,**R**) NH_4_^+^. (**A**,**C**,**E**,**G**,**I**,**K**,**M**,**O**,**Q**) nodulated plants; (**B**,**D**,**F**,**H**,**J**,**L**,**N**,**P**,**R**) non-nodulated plants. Treatments: Cont, control with N-free solution; NO_3_^−^, 5 mM KNO_3_; NH_4_^+^, 2.5 mM (NH_4_)_2_SO_4_; urea, 2.5 mM urea; and NO_3_^−^ + NH_4_^+^, 2.5 mM KNO_3_ + 1.25 mM (NH_4_)_2_SO_4_. *n* = 3. Average ± standard error. Different letters on the top of the columns indicate significant differences in the N concentrations among the organs based on Tukey’s test (*p* < 0.05).

The concentrations of N metabolites among the organs with the various N treatments were compared (Figure 8). The nitrate concentrations in the Nod and Non-nod plants were significantly higher in the lower roots compared with the upper roots when 5 mM NO_3_^−^ was supplied for 1 day (Figure 8A,B). The nitrate concentrations in the stem, leaves, and nodules were much lower than in the upper and lower roots. Similar trends were observed when NH_4_^+^ + NO_3_^−^ was applied, although the NO_3_^−^ concentrations were lower than NO_3_^−^ treatment.

Figure 8C–F shows a comparison of the concentrations of ureides, allantoate, and allantoin, among the organs of the Nod and Non-nod plants. In the Nod plants, the concentration trends affected by the N treatments were relatively similar between allantoate and allantoin, although the concentration of allantoate was higher than allantoin. The highest concentration of allantoate and allantoin was observed in the stems, followed by the leaves, nodules, upper roots, and lower roots. The N-treatments did not change the concentration patterns of allantoate and allantoin in the Nod plants. The concentrations of allantoate and allantoin in the Non-nod plants were not different among the organs, except for the allantoate concentration in the lower roots with the urea treatment.

Figure 8G–J shows the concentrations of amides, Asn, and Gln among the organs. In the Cont Nod plants, the concentration of Asn was significantly higher in the stems. When NO_3_^−^ was applied, the concentration of Asn in the lower and upper roots increased. When NH_4_^+^ or urea was supplied to the culture solution, the concentrations of Asn, especially in the lower and upper roots, increased. The trends in the concentrations of Gln among the organs were similar to those of Asn, although the concentrations were lower than Asn. The concentration of Asn in the Non-nod plants was low in the control treatment, and the NH_4_^+^ or urea application increased the Asn concentration in the lower and upper roots. The applications of NH_4_^+^ or urea increased the concentrations of Gln, especially in the upper roots.

Figure 8K–N shows a comparison of the concentrations of the major amino acids, Asp and Glu, among the organs. In the nodules of the Nod plants with the Cont, the Asp and Glu concentrations were significantly higher than the other organs. The N treatments did not alter the Asp and Glu concentrations. The concentrations of Asp and Glu in the Non-nod plants were relatively lower than those in the Nod plants, and the Asp concentrations were not significantly different among the organs. On the other hand, the Glu concentration was higher in the leaves in the Non-nod plants, and the N treatments tended to increase the Glu concentration in the leaves.

The urea concentration was significantly higher in the stems compared with the lower and upper roots, leaves, and nodules in the Cont treatment of the Nod plants (Figure 8O,P). The trends were the same in the Nod plants with the various N treatments. On the other hand, the urea concentrations in the stem of the Non-nod plants were not higher than in the other organs.

As for the ammonium concentrations in the Nod plants with the Cont treatment, the nodules showed an approximately 10-fold higher concentration than the other organs (Figure 8Q,R). When NH_4_^+^ and NO_3_^−^ + NH_4_^+^ were the treatments, the ammonium concentrations in the upper and lower roots became higher than in the Cont treatment. In the Non-nod plants, the applications of NH_4_^+^, urea, and NO_3_^−^ + NH_4_^+^ enhanced the ammonium concentrations in the lower and upper roots.

## 4. Discussion

### 4.1. N Metabolites in the Non-Nodulated Plants

In this research, the effects of nodules on the absorption, assimilation, and transport of N in roots supplied with various N forms were compared. The Nod plants were cultivated with an N-free solution and depended on sole N_2_ fixation. The Non-nod plants were precultivated in a solution with 1 mM NO_3_^−^ to support the N demand for growth. For 3 days before the N treatments (from 27 to 30 DAP), the culture medium was changed to an N-free solution to decrease the concentrations of NO_3_^−^ and major N metabolites in the Non-nod plants. The concentrations of NO_3_^−^ and N metabolites in the xylem sap collected from the Cont Non-nod plants were low due to the fact of the N starvation for 4 days. Low levels of NO_3_^−^ were occasionally detected in the plant parts cultivated with an N-free solution, possibly due to the fact of contamination from the solution, reagents, or environment [18].

Although the concentrations of N metabolites in the Cont treatment were low, small amounts of allantoate, urea, Gln, Asp, Asn, and Glu were detected. The concentration of allantoate was significantly lower in the Non-nod plants compared with the Nod plants, but allantoate was detected in the xylem of the Cont Non-nod plants. Ureides are universal metabolites in animals, plants, and microorganisms produced by purine degradation. In plants, these compounds play a role in the storage and translocation of N in several species, such as maple (*Acer saccharum*) and comfrey (*Symphytum officinale*) [20,21]. It is established that ureides are the principal compounds that transport N from the soybean nodules to the shoot via the xylem vessels. The economically important legume crops, such as soybeans, beans (*Phaseolus vulgaris* L.), and cowpeas (*Vigna unguiculate*), are ureide-transporting plants [22]. Ureides in the Non-nod xylem sap might be derived from the degradation or turnover of the nucleic acids and protein in the roots.

In the NO_3_^−^ treatment, the concentrations of NO_3_^−^ and Asn in the xylem sap increased significantly, although the other amides and amino acids did not change. In the NH_4_^+^ treatment, the concentrations of Asn and Gln increased significantly compared with the Cont. Because the concentrations of ureides did not increase due to the NH_4_^+^ supply, ureides synthesis in the roots was not promoted by 1 day of the ammonium supply. Ammonium could not be detected in the xylem sap in the NH_4_^+^ treatment. It is known that NH_4_^+^ is toxic as well as nutritious, and the absorbed ammonium should be quickly assimilated into nontoxic compounds, such as Asn and Gln, in the roots and transported via the xylem. In the urea treatment, the concentrations of Asn, Gln, and Asp increased, but the other amino acids and ureides did not. Yamashita et al. [2] reported that the nodulated soybean roots absorbed ^15^N-labeled urea and assimilated and transported it to the shoots.

The concentration of urea in the xylem sap with the urea treatment did not increase, so the urea absorbed in the roots might have been degraded to ammonium in the roots by the enzyme urease and assimilated to Gln and Asn in a similar manner to under the NH_4_^+^ treatment. In the NO_3_^−^ + NH_4_^+^ treatment, the concentration of NO_3_^−^ was half of that in the NO_3_^−^ treatment, possibly due to the suppression of the nitrate transporter or NR by the copresence of NH_4_^+^. The NO_3_^−^ + NH_4_^+^ treatment increased the Asn and Gln concentrations as did the NH_4_^+^ and urea treatments but not for ureides.

The effects of the N treatments on the lower roots (Figure 5B) and the upper roots (Figure 4B) were similar to the concentration changes in the xylem sap of the Non-nod plants. The xylem sap compositions might reflect the roots’ metabolism. The effects of the N treatments on the stems (Figure 6B) and leaves (Figure 7B) were similar to the xylem sap composition, but the Asn concentrations in the leaves and stems were the highest in the NO_3_^−^ + NH_4_^+^ treatment.

### 4.2. N Metabolites in Nodulated Plants

In the Nod plants with Cont treatment, ureides were the principal N metabolites analyzed, and allantoate concentration was about two-fold higher than allantoin irrespective of the treatments. The percentages of N metabolites analyzed were allantoate 58%, allantoin 30%, Asn 8%, Gln 3.4% in the xylem sap of the Nod plants. This result agreed with the previous reports that up to 90% of the fixed nitrogen is transported to the shoots as the ureides, allantoin, and allantoate [22,23].

In the NO_3_^−^ treatment for 1 day, the concentrations of allantoin and allantoate significantly decreased compared with the Cont xylem sap. Ono et al. [15] reported that 3 days of 5 mM NO_3_^−^ treatment decreased the ureides concentration in the xylem by approximately one-seventh of the control plants. The NH_4_^+^ treatment increased the concentrations of Asn and Gln but did not affect the allantoate and allantoin concentrations (Figure 2A). The urea and NO_3_^−^ + NH_4_^+^ treatments increased the Asn and Gln concentrations but tended to decrease the concentrations of ureides.

The N metabolite concentrations in the nodules were not affected by the N treatments, except for NO_3_^−^. The concentration of NO_3_^−^ in the nodules treated with 5 mM NO_3_^−^ was approximately 500 mg N g^−1^DW and much lower than those in the lower roots (3900 mg N g^−1^DW) and the upper roots (2900 mg N g^−1^DW). It was reported that NO_3_^−^ is absorbed from the nodule surface [24], but the absorption and accumulation of NO_3_^−^ in the nodules were much lower than in the roots. In the lower roots of the Nod plants, the NH_4_^+^ and urea treatments increased the concentrations of Asn, Gln, and allantoate. It is noteworthy that the ammonium and urea treatments enhanced the concentration of allantoate in the lower roots significantly compared with the Cont plants. In the upper roots, the NH_4_^+^ and urea treatments increased the concentrations of Asn and Gln but did not affect the allantoate concentration. In the stems (Figure 6) and leaves (Figure 7), the concentrations of Asn and Gln increased due to the N treatments, but the concentrations of allantoate and allantoin were not significantly affected.

The urea concentrations in the stems of the Nod plants were significantly higher than those in the Non-nod stems (Figure 8). Ono et al. [15] reported that some of the ureides produced in the nodules might degrade into urea in the roots. However, the results of this experiment suggest that some ureides degraded to urea in the stems. Urea is a major N form supplied as fertilizer and is an important N metabolite in plants [25]. Mérigout et al. [25] reported the ^15^N-urea uptake in *Arabidopsis thaliana* stimulated by urea but reduced by the presence of ammonium nitrate in the growth medium. Yamashita et al. [2] reported that ^15^N-urea was efficiently absorbed in soybean roots and transported to the shoots. Two types of plant membrane transporters, namely, major intrinsic proteins (MIPs) and the DUR3, were shown to play roles in low- and high-affinity urea transport, respectively [26]. Although urea is a ubiquitous metabolite in plants and is synthesized either by purine degradation or arginine metabolism, the major metabolic pathway and sites of urea synthesis in soybean are still controversial. Ono et al. [15] suggested that a major part of the urea in nodulated soybean plants might originate from ureide degradation. In addition, urea production may occur in roots based on the finding that a positive correlation was observed between the concentrations of ureides and urea in xylem sap, as well as in the leaves and roots, but the correlation efficiencies between urea and arginine were low. In this report, urea was present in the xylem sap and all organs of the Non-nod plants, but 1 day of N supply did not increase the urea concentration in the lower roots.

### 4.3. N Metabolites among Organs

The higher nitrate concentrations in the lower roots compared with the upper roots might reflect the higher NO_3_^−^ absorption activity in the lower roots than the upper roots (Figure 8A,B). The lower concentration of NO_3_^−^ in the NH_4_^+^ + NO_3_^−^ treatment compared with the NO_3_^−^ treatment might be due to the fact of a lower concentration of NO_3_^−^ or repression of NO_3_^−^ absorption by coexisting NH_4_^+^ [27].

Figure 8C–F show a comparison of the concentrations of ureides, allantoate, and allantoin in each part of the Nod and Non-nod plants. In the Nod plants, most of the ureides should have been synthesized in the root nodules, but the concentrations of allantoate and allantoin were lower than in the stems.

Interestingly, the concentrations of allantoate in the Non-nod plants increased in the lower roots with the urea treatment, suggesting that this condition might enhance the allantoate synthesis. Yoneyama et al. [28] reported that the concentrations of ureides in the leaves and stems were higher in the nodulated and non-nodulated soybean plants supplied with urea compared with N-free conditions. The direct condensation of urea and glyoxylate was denied in early studies [29].

Amides, Asn, and Gln were the major N forms from the absorbed NH_4_^+^ or urea in the roots. NH_4_^+^ is a toxic compound, so the absorbed NH_4_^+^ should be assimilated quickly. The concentrations of NH_4_^+^ were relatively low in each organ and were not detected in the xylem sap when NH_4_^+^ was supplied to the solution. After 1 day of NO_3_^−^ treatment, the concentrations of Asn in each organ were not high, but after 3 days of NO_3_^−^ application, the Asn concentration was very high [15]. In *Arabidopsis*, the ^15^N urea and ^15^N ammonium assimilation pathways are similar [25]. The soybean plants supplied with urea and NH_4_^+^ were essentially the same in this experiment as in former reports [2,15].

### 4.4. Effects of Nodulation on N Absorption, Metabolism, and Transport

Similar changes due to the N treatments were observed between the nodulated and non-nodulated soybeans, suggesting that nodulation did not have significant effects on the metabolism and transport of the absorbed N in the roots. The inhibitory effects of the combined N on nodule growth and N_2_ fixation activities have been reported [2,16], and this was attributed to a decrease in the photoassimilate supply in the nodules by the increased photoassimilate consumption in the roots with N absorption and assimilation [16]. The soybean plants give priority to the N absorption from the roots over N_2_ fixation by nodules. It was reported that when N was supplied in the medium, over 3 weeks of the 5 mM NO_3_^−^ treatment, the nodule growth almost stopped and N_2_ fixation tentatively decreased, but the nodules’ growth and N_2_ fixation activity quickly recovered when the solution was changed to an N-free solution [30,31]. Therefore, the nodules did not disintegrate but stayed inactive when N was supplied from the medium.

## 5. Conclusions

To investigate the effects of nodulation on the N metabolism of soybean plants supplied with various N forms, the concentrations of the principal N metabolites in the xylem sap and each organ were compared between the nodulated and non-nodulated soybean plants after 1 day of treatment with NO_3_^−^, NH_4_^+^, urea, and NO_3_^−^ + NH_4_^+^. Allantoate and allantoin were the principal N compounds, and the sum was 90% of the total N analyzed in the xylem sap of the nodulated plants with an N-free solution. The concentrations of the N metabolites in non-nodulated plants cultivated with an N-free solution were lower than those in the nodulated plants, and changes in the N metabolite concentrations after the N treatments were essentially the same as those in the nodulated plants. Similar changes due to the N treatments were observed between the nodulated and non-nodulated soybeans, suggesting that nodulation did not have significant effects on the metabolism and transport of the absorbed N in the roots. The concentration changes in the lower roots, upper roots, stems, and leaves were relatively similar to the changes in the xylem sap compositions after 1 day of the N treatment. The NO_3_^−^ and NO_3_^−^ + NH_4_^+^ treatments increased the NO_3_^−^ concentrations in the lower roots and the upper roots, whereas the NH_4_^+^ and urea treatments promoted the accumulation of Asn and Gln.

## Data Availability

Not applicable.
